# The effect of incidental name similarity on favoritism in the Chinese financial market

**DOI:** 10.1038/s41598-025-97364-x

**Published:** 2025-04-16

**Authors:** Kaixian Mao, Huidi Lu, Shirley Jiexuan Wang

**Affiliations:** 1 Renmin University of China, Beijing, 100872 China; 2 Saïd Business School, University of Oxford, Oxford, OX1 1HP UK; 3 Nankai University, Tianjin, 300192 China

**Keywords:** Incidental similarity, Behavioral decision making, Favoritism, Social interactions, Financial analysts, Earnings forecast, Human behaviour, Psychology

## Abstract

Incidental similarities between individuals can sway professional judgment, sometimes resulting in people overstepping legal or ethical boundaries. Recent research on the US financial sector indicates that incidental name similarities between CEOs and securities analysts induce favoritism and lead to more accurate earnings forecasts, likely by facilitating unfair private information disclosure. This study investigates similar phenomena in China using richer datasets, including records of face-to-face interactions between analysts and company executives during corporate site visits. Interestingly, while the results confirm that Chinese analysts who share surnames with company executives exhibit favoritism, this favoritism manifests as overly optimistic earnings forecasts after corporate site visits. This is particularly notable with less common surnames. Our findings highlight the cultural specificity of surname-driven biases in social interactions.

## Introduction

Name similarity, a trait incidentally shared between individuals, has been shown to exert a strong influence on judgment and decision-making. Across various domains, from consumer behavior to interpersonal relationships, name similarity can foster feelings of affinity and preferential treatment^1,2^. These effects are part of a broader set of phenomena where incidental similarities, such as sharing the same birthday or hometown, significantly shape how individuals perceive and interact with one another^3–7^. In the context of high-stake financial decisions, the impact of incidental similarities, particularly name similarity, has gained attention for its potential to bias professional judgments^8,9^.

Financial analysts play a critical role in gathering corporate information and providing investment advice^10^. Their forecasts and recommendations can influence multibillion-dollar investments in the financial sector. Research suggests that personal similarities, such as shared surnames between analysts and corporate executives, may enhance rapport and facilitate information sharing, potentially reducing information asymmetry and improving forecast accuracy^9,11–13^.

However, most of these studies are based on US data only. Despite the documented effects of incidental similarities, few studies show their variability across different cultural contexts, where different social values can shape interpersonal interactions^14^. One notable example investigates the impact of incidental name similarity on financial analysts’ earnings forecasts^9^. Analysts in the US, who share the same first name with executives of S&P 1500 firms, have been found to issue more accurate earnings forecasts of those firms. This effect is more pronounced when the shared first name is rare and diminishes as the name is more common. The authors posit that individuals have an affinity for those who share first names, which can manifest itself in the CEO sharing private information with a matched analyst. While the study provides invaluable insights, it remains unclear whether their findings extend to other cultural contexts.

Our study tries to supplement this line of research by uncovering a contrasting dynamic in a different cultural context: analysts in China who share surnames with the company executives tend to issue *less* accurate but overly optimistic earnings forecasts, especially after face-to-face interactions with company executives. This sheds light on the role of cultural factors in shaping the impact of incidental similarities on information in the financial market. Similarly, resonating with the US-based study^9^, we find stronger effects when the shared surname is rare.

In this study we focus on surnames because sharing the same first name is less common in China compared to in the US^15,16^. Sharing the same surname in China is much more common, and carries social and cultural underpinnings^17^. According to the Home Affairs Center of the Chinese Ministry of Public Security^18^, as of 2018, there are 6150 different surnames in use by Chinese citizens, and the top 100 surnames cover about 85% of the total population. A balanced distribution of the population across various surnames makes surname coincidences surprising enough, while also providing us with sufficient statistical power to detect any potential surname effect. Furthermore, Chinese women do not adopt their husband’s surname after marriage. Thus, we avoid potentially confounding influences from analysts’ marital status changes.

Incidental surname similarity may bring about different consequences on people’s judgement and decision making in China because the culture emphasizes kinship-based relationships^19^ and enhancing each other’s reputation and social standing (i.e., *Mianzi*) is crucial in such societies^20–22^. First, different from the US, Chinese people value greatly the symbolic linkage implied by their surnames^23,24^. Surnames often represent important ancestral information and serve as symbolic connections between individuals and their progenitors^25–27^. In China, surnames are usually passed down from the father and represent a part of an individual’s genetic heritage^27^. In this context, two individuals sharing the same surname will not only perceive each other as similar but also consider themselves to have the same ancestors^28^. This broader and subtler association between the two individuals can induce stronger favoritism, potentially contaminating rational judgement even in professional settings.

Second, cultural values such as *Mianzi*—the concept of face and social reputation in Chinese society—are deeply embedded in historical and social contexts. According to cultural evolutionary theory^29,30^, social values like *Mianzi* are shaped by recurring social and environmental challenges, gradually influencing cognitive and emotional responses over time. Social values function on multiple levels—both mentally and emotionally—and are deeply embedded within specific cultural frameworks^31^. In the case of *Mianzi*, these values reflect longstanding cultural norms that guide decision-making and social behavior, contrasting sharply with individualistic values such as autonomy, which are more prevalent in Western societies.

Importantly, the motivation to enhance *Mianzi* tends to arise within networks of people who know each other and share established relationships. This contrasts with mere favoritism, as the emphasis on *Mianzi* emerges from mutual obligations and social ties within broader family or social networks. These theories highlight how values like *Mianzi* emerge from unique historical and societal pressures, reinforcing their role as a culturally specific motivator of behavior, rather than a universal social norm.

For analysts, the cultural emphasis on *Mianzi* may lead to overly optimistic forecasts, driven by the need to protect or elevate the social standing of executives with whom they share a surname, sometimes in exchange for favors. In addition to ensuring forecast accuracy, analysts are compelled to safeguard the *Mianzi* of company executives, perceiving them as part of a broader family or social network. Indeed, previous research indicates that Chinese professionals sometimes even engage in unethical behavior to protect or enhance the *Mianzi* within their relational circles^32,33^. Therefore, these analysts may produce overly optimistic forecasts to improve the financial appearance of the companies and bolster the *Mianzi* of the executives, not merely out of favoritism but as part of a different cultural obligation tied to their relationships.

Overall, this study contributes to a broader conversation on the influence of incidental similarity. We demonstrate how incidental surname matching between analysts and corporate executives lead to different outcomes in different cultural contexts. Our results supplement previous findings in the US and highlight the role of culture in understanding the behavioral implications of incidental similarities.

In the following sections, we first describe the data source and analytical strategy. We then demonstrate the results of our analysis. First, we show that Chinese analysts produce significantly over-optimistic earnings forecasts following interactions with executives of the company who happen to share the same surname. Second, we show that this effect maintains, albert being weaker, among *all* analysts who share the same surname with executives of the company they are covering, even in the absence of face-to-face interactions. We then provide evidence that the name similarity effect is stronger when the matched surname is rare. We conclude by discussing the theoretical contributions to studies of incidental similarity and the practical implications for market regulation.

## Data sources

Our data come from the Chinese Stock Market and Accounting Research Database^34,35^ (CSMAR, https://data.csmar.com). We obtain company fundamentals and the full history of analyst forecast reports and analyst site visits from 2011 to 2019. We are able to trace the complete sample of corporate site visits to Chinese listed firms thanks to a regulatory request that all site visits of security analysts be disclosed by the listed companies since 2009. However, we do not extend our sample after the COVID-19 outbreak in 2019 because offline site visits were paused in China during the pandemic due to strict mobility control measures.

Our main variables are calculated based on the analyst forecast data. The dataset records the release date of a forecast report, the names of the analysts writing the report and their characteristics including years of experience, and the forecasted EPS. One forecast report may contain multiple forecasts of the same company with different horizons (EPS in one year, in two years, etc.). Combined with the end-year realized EPS and daily stock price data, we compute the accuracy and optimism of each forecast.

Additionally, based on the corporate site visits data, we have the names and affiliations of all visitors (including the analysts), names of the hosting staff who are usually the company chief officers or the board secretary, as well as dates of the visits. Using these records, we identify analysts who issued forecast reports after face-to-face interactions with company executives. The availability of analyst forecasts and site visits records makes China’s A-share listed market a unique setting to analyze how favoritism introduced by incidental name similarity during social interactions may affect professional judgements.

## Measures

We describe the variables involved in our analysis below. Summary statistics of these variables are available in the Supplementary Material, Part SM1.

### Dependent variable 1: relative forecast accuracy (RFA)

Following previous research^9^, we first look at the relative forecast accuracy (RFA) as our key dependent measure. This variable measures how accurate an analyst’s earnings forecast is, relative to the average forecast error of other analysts covering the same firm. A higher RFA indicates that the analyst’s forecast is more accurate than others. For each analyst *i* covering firm *j* in year *t*, we first calculate the earnings forecast error, *FE*_*ijt*_, which equals the difference between the forecasted value and the realized earnings for the specific year. The analyst’s relative forecast accuracy, *RFA*_*ijt*_, is calculated as:

1 $$\begin{array}{c}RF{A}_{ijt}=\frac{\left|\overline{F{E}_{jt}}\right|-\left|F{E}_{ijt}\right|}{\left|\overline{F{E}_{jt}}\right|},\end{array}$$

where $\left|F{E}_{ijt}\right|$ is the absolute value of *FE*_*ijt*_ and $\left|\overline{F{E}_{jt}}\right|$ is the average of absolute forecast errors of all analysts who followed the same firm in the same year.

### Dependent variable 2: forecast optimism (ROPT)

Forecast optimism (ROPT) captures how optimistic an analyst’s forecast is relative to other analysts^36^. A higher ROPT indicates that the analyst overestimated the firm’s earnings compared to others. The relative forecast optimism, *ROPT*_*ijt*_, of analyst *i* covering firm *j* in year *t* is given by:

2 $$\begin{array}{c}ROP{T}_{ijt}=\frac{F{E}_{ijt}-\overline{F{E}_{jt}}}{\left|\overline{F{E}_{jt}}\right|}.\end{array}$$

A higher *ROPT*_*ijt*_ implies stronger optimism in the analysts as reflected in the forecasts they produce.

### Independent variables

Our primary independent variable of interest, *surname match*, is an indicator variable corresponding to whether the analyst has the same surname as the actual hosting person (usually the chief officers or the board secretary) during site visits. Additionally, closely following similar research in the U.S. setting^9^, we include several control variables in our regression models. *Forecast horizon* is the number of days between the issuing date of the forecast report and the release date of the actual earnings. Analyst *experience* is the normalized work experience (in quarters) of the analyst at the time of making the forecast by deducting the average work experience of other analysts who also made forecasts on the same company in the previous 60 days. *Number of firms following* is the normalized total number of companies on which the analyst issued forecasts in the same year by deducting the average number of companies followed by other analysts who also made forecasts on the same company in the previous 60 days. Following studies on the analyst forecasts, we control for various combinations of fixed effects to capture unobserved time-invariant, firm-specific or analyst-specific factors that could influence the analyst forecast accuracy. In the most stringent empirical design, we also control for analyst-year fixed effects to account for time-varying factors that may influence individual analysts’ performance.

Finally, we control for *uncommon surname*, constructed as a binary variable aligning with previous U.S. study^9^, which is equal to one if the analyst’s surname is classified as uncommon and is equal to zero otherwise. We define surnames as uncommon if it is used by fewer than 0.5% of the population based on the Chinese census^9,37^. This threshold is different from the U.S. study (in which the authors use 2% to identify uncommon first names) due to the different distribution of surnames in China. We describe the detail and conduct several robustness tests on the threshold in Supplementary Materials, Part SM 2. We control for the uncommonness of the surnames due to the following reasons. First, previous studies find that it can affect how individuals are perceived and formations of human and social capital^38–40^, which may as well influence the accuracy of financial forecasts due to information (dis)advantages. Second, considering that hiring process might also consider surname rareness^9^, financial analysts with rare surnames may perform inherently better than financial analysts with common surnames, an effect which we would like to disentangle. Third, we aim to account for the likelihood that surname-matching are more probable among individuals with common surnames, similar to Even-tov et al.^9^ in their U.S. study on common first names. Thus, our main regression design include the uncommonness of the analyst’s surname.

## Results

### Overly optimistic forecasts by analysts following interactions with company executives sharing the same surname

Our study is motivated by the hypothesis that analysts in China may respond differently to incidental name similarity compared to those in the U.S., leading to misjudgments rather than reducing information asymmetry. This tendency may be particularly pronounced in contexts involving face-to-face interactions, where cultural norms like *Mianzi* and social affinity are more salient. To examine Chinese analysts’ responses, we use differences in forecasts among financial analysts who took part in the same corporate site visit to uncover the impact of sharing the same surname with company executives. We do so by keeping only the first issue of analyst forecast reports within 30 days after a site visit. We also restrict our sample to site visits with at least two analysts where at least one analyst who shares the same surname with the reception staff, and at least one who does not, to control for the site-visit fixed effects in our regressions (Columns 1 and 3 of Table 1). Visit-specific effects control for many other covariates such as information contents in that visits that may confound our focal surname effect. Any time-specific effects (such as the season or weather) are ruled out because analysts join the same visit at the same time. Firm-specific effects (such as the listed firm’s financial fundamentals) are also absorbed as visits are specific to one company. We also control for analyst fixed effects that could play a role in the analyst forecasting process. For robustness, in a different specification, we control for firm fixed effects and analyst time-varying attributes (Columns 2 and 4 of Table 1). We refer to the Supplementary Material, Table SM1 Panel A, for summary statistics of the variables in this sample.

**Table 1:** Effect of surname match during site visits on forecast accuracy and optimism.

	(1)	(2)	(3)	(4)
	RFA	RFA	ROPT	ROPT
Surname match	− 1.4***	− 0.36***	0.40**	0.31***
	(− 9.27)	(− 24.49)	(2.12)	(18.39)
Forecast horizon	− 0.00022**	− 0.0002*	0.00015	0.00015
	(− 2.03)	(− 1.91)	(1.64)	(1.60)
Experience	− 0.87***		1.2***	
	(− 4.49)		(4.92)	
# Firms following	− 0.21***		0.35***	
	(− 21.27)		(29.03)	
Constant	− 2.4***	0.14***	4.2***	− 0.096**
	(− 7.79)	(2.85)	(11.07)	(− 2.19)
Visit FE	Yes	No	Yes	No
Analyst FE	Yes	No	Yes	No
Firm FE	No	Yes	No	Yes
Analyst-year FE	No	Yes	No	Yes
*N*	802	818	802	818
*R*^2^	0.655	0.654	0.790	0.790

Continuous variables are winsorized at the 99th percentile. T-statistics are displayed in parentheses. Robust SEs are clustered at the firm level. Statistical significance *p < 0.1, **p < 0.05, ***p < 0.01.

Results in Table 1 suggest that sharing the surname with company executives is significantly associated with lower forecasting accuracy and higher forecasting optimism. Two specifications in Column 1 (b = − 1.4, t = − 9.27, *p* = 0.00) and Column 2 (b = − 0.36, t = − 24.49, *p* = 0.00) yield consistent results. They indicate that sharing the same surname with the executive can sometimes double the relative error of the analyst. Column 3 (b = 0.4, t = 2.12, *p* = 0.03) and Column 4 (b = 0.31, t = 18.39, *p* = 0.00) also yield similar results and show that sharing the same surname with company executives increases an analyst’s relative optimism by over 30%.

The above results are consistent with our hypotheses of the main effect and hint on the tendency that Chinese analysts produce over-optimistic forecasts for companies whose executives share the same surnames with them. These results highlight a different dynamic when compared to the US context. In China, surname matching appears to amplify optimism rather than improve accuracy^9^, suggesting that incidental similarity effect is highly dependent on the culture background. To ensure the robustness of our findings, the subsequent section extends the analysis to include all analysts who share a surname with company executives, irrespective of face-to-face interactions.

### Overly optimistic forecasts by analysts who share the same surnames as the CEOs

In this section, we relax the condition of face-to-face interactions during site visits and extend our analysis to the universe of all financial analysts and their forecast reports. We redefine the independent variable, *surname match*, as whether the analyst has the same surname as the CEO of the covered firm in the reporting year. This approach parallels studies conducted in the U.S.^9^, which investigated the effects of sharing the same first name with the CEO on analyst forecasts.

We use linear regressions to test the effect of sharing a surname with corporate CEOs on Chinese analysts’ forecast accuracy (RFA) and optimism (ROPT). We follow the prior literature and retain just the last forecast by an analyst for a specific company each year^9^. To control unobservable factors affecting analysts’ forecasting accuracy, we also include year and firm fixed effects (Columns 1 and 3, Table 2). We also estimate an uncontrolled model accounting for both analyst-year and firm fixed effects (Columns 2 and 4, Table 2), similar to the previous US study^9^. This gives us 399,759 observations in total. Sample sizes may reduce in estimations depending on availability of the independent variables and removal of singleton groups that can bias the estimation results^41,42^. We refer to the Supplementary Material, Table SM1 Panel B, for summary statistics of the variables in this sample.

**Table 2:** Effect of surname match on forecast accuracy and optimism.

	(1)	(2)	(3)	(4)
	RFA	RFA	ROPT	ROPT
Surname match	− 0.00027	− 0.0021	0.0069*	0.0074**
	(− 0.09)	(− 0.70)	(1.84)	(2.04)
Forecast horizon	− 0.00037***	− 0.00022***	0.0003***	0.00017***
	(− 79.45)	(− 66.07)	(43.00)	(36.30)
Experience	0.00048***		− 0.00063***	
	(2.73)		(− 2.78)	
# Firms following	− 0.000029		− 0.000026	
	(− 0.61)		(− 0.44)	
Uncommon surname	0.0056**		− 0.0074**	
	(2.01)		(− 2.09)	
Constant	0.22***	0.16***	− 0.18***	− 0.13***
	(87.10)	(96.71)	(− 52.73)	(− 58.70)
Year FE	Yes	No	Yes	No
Firm FE	Yes	Yes	Yes	Yes
Analyst-year FE	No	Yes	No	Yes
*N*	358,996	397,822	358,996	397,822
*R*^2^	0.048	0.213	0.030	0.215

Continuous variables are winsorized at the 99^th^ percentile. T-statistics are displayed in parentheses. Robust SEs are clustered at the firm level. Statistical significance *p < 0.1, **p < 0.05, ***p < 0.01.

Results in Table 2 suggest that forecast accuracy is again lower for analysts whose surname is the same as the CEO, although such effects are not significant (Columns 1 and 2, Table 2). However, we do find that analysts are significantly more optimistic when the surnames match. Two specifications in Column 3 (b = 0.0069, t = 1.84, *p* = 0.06) and Column 4 (b = 0.0074, t = 2.04, *p* = 0.04) in Table 2 yield similar results. They indicate that simply sharing the same surname with the CEO increases an analyst’s relative optimism in financial forecasts by around 0.7%. Thus, we believe that our empirical findings are in line with the argument that incidental surname similarities lead to optimistically biased financial forecasts in China. We note the pattern is weaker, likely because merely knowing there is a surname match does not evoke a strong sense of affinity. And the intent to enhance the *Mianzi* of the company executive arises only after establishing a relationship through face-to-face interactions. This again confirms that the effect of incidental similarity can manifest differently in different cultures.

We further conducted an online experiment to verify the cultural specificity of incidental name similarity effects. This experiment, pre-registered on *AsPredicted* (https://aspredicted.org/kkb2-z2s3.pdf), used a 2 (Nationality: China vs. US) × 2 (Surname Match vs. No Information) between-subjects design. The 2 × 2 between-subjects design allows us to isolate the effects of surname matching and cultural context, making it possible to compare perceptions across different nationalities and name conditions. We recruited 220 Chinese participants on Credamo and 220 Americans in the US via Prolific. These participants were randomly assigned to either the surname match condition, where they are told that they just met a person of the same surname as theirs, or to the no information condition, where surname of the other person was not mentioned. We then asked about four main aspects: (1) perceived familial connection, (2) perceived value similarity, (3) willingness to exchange contact information, and (4) likelihood of forming a friendship, using a 1–5 Likert scale. We obtained ethical approval from the School of Labor and Human Resources ethical committee at the Renmin University of China (ID: RUC-SLHR20240011). All procedures followed relevant ethical guidelines, and informed consent was obtained from all participants.

**Fig. 1:**
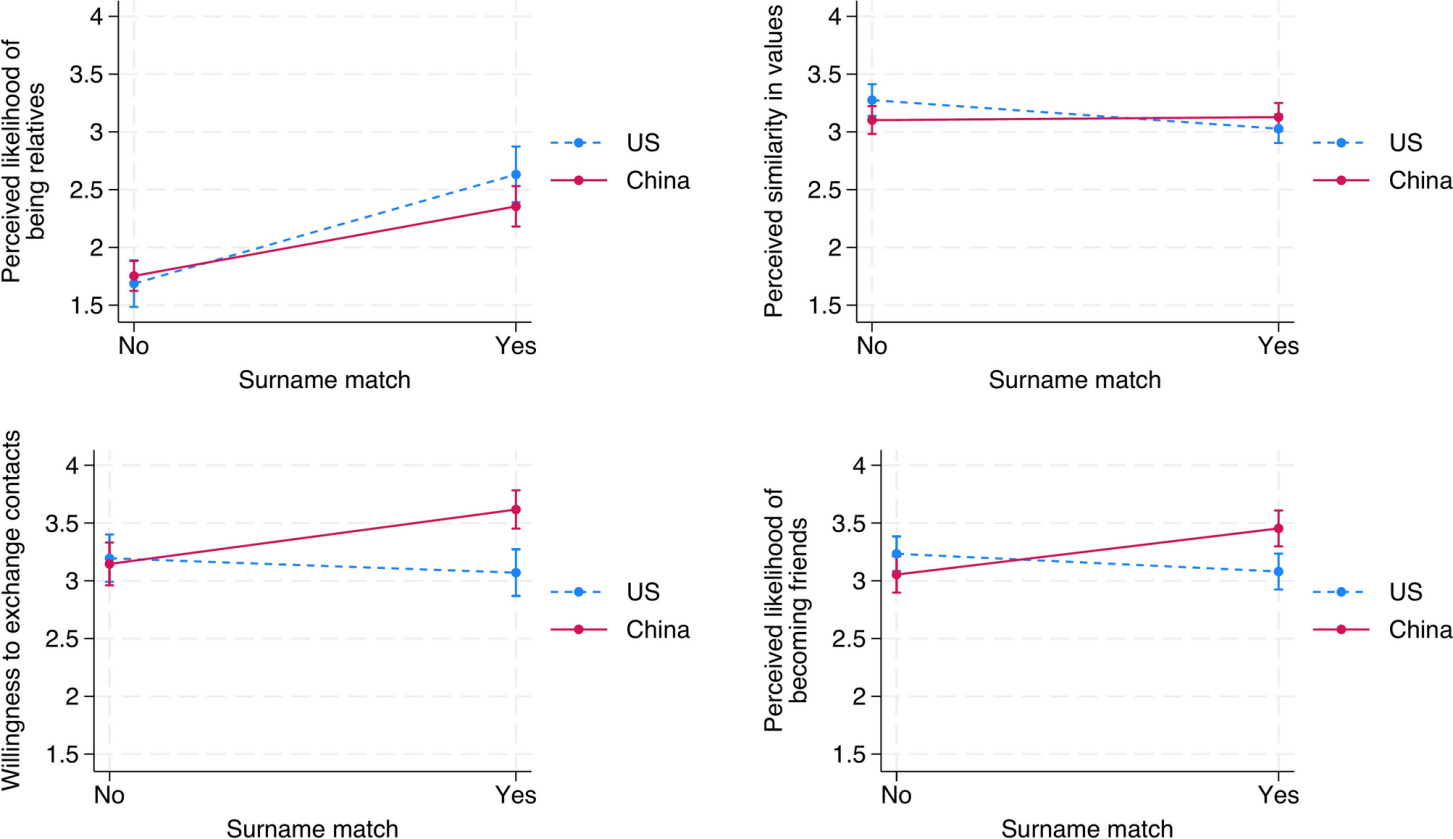
Surname match effect on perceived relationships and social outcomes in Chinese and US respondents.

Our experimental results (*N* = 432) shows that while both Chinese and Americans understand the genetic or familial link imbedded in surnames, Chinese respondents reacted more strongly to surname matching compared to US respondents. Importantly, as shown in Fig. 1, Chinese participants were more likely to believe they share similar values to someone who shared their surname (*p* < 0.05), were more willing to exchange contact information (*p* < 0.01) and form friendships (*p* < 0.01) based on surname similarity. This further highlights the cultural significance of surnames in professional and social interactions. Detailed regression results are available in Supplementary Material, Part SM3.

### Stronger effects for less common surnames

Our analyses further reveal that the effect of surname sharing on forecasts is more pronounced for analysts with less common surnames. Chinese surnames differ in their distribution for historical reasons^17^. Some family names are very common, while others are used by very few individuals. This can affect the surname-sharing effect on analyst bias. The surprising value of a rare surname coincidence will trigger the self-enhancement process more heavily. The rarity of a surname therefore enhances the perceived connection between the analyst and the executive, leading to a stronger emotional response and, consequently, greater optimism in earnings forecasts.

We present our empirical findings results in Table 3, using the model specification similar to the previous section but dividing the main independent variable into (1) one indicator variable representing whether the analyst and the executive shared a surname defined as common and (2) another indicator variable representing whether the analyst and the executive shared a surname defined as uncommon^9^. We see the tendency that when shared surnames are rarer, the analyst will be more biased toward the company. In particular, in Column 3 of Table 3, sharing an uncommon surname increases forecast optimism by 0.70 whereas sharing a common surname increases optimism by 0.36. The difference is statistically significant (*p* = 0.03). In Column 4, sharing an uncommon surname also increases forecast optimism more than a common surname (0.45 vs. 0.31, *p* < 0.01). These results are robust if we use different thresholds to construct the uncommon surname indicator (Supplementary Material, Part SM2) or use an alternative interaction model (Supplementary Material, Part SM4).

**Table 3:** Effect of matched surname commonness on forecast accuracy and optimism.

	(1)		(2)		(3)		(4)	
	RFA		RFA		ROPT		ROPT	
Surname match—common	− 1.5***	*p* = 0.15	− 0.36***	*p* = 0.64	0.36*	*p* = 0.03**	0.31***	*p* = 0.0096***
	(− 8.87)		(− 24.46)		(1.89)		(18.37)	
Surname match—uncommon	− 1.3***		− 0.27		0.70***		0.45***	
	(− 20.13)		(− 1.47)		(11.36)		(7.69)	
Forecast horizon	− 0.00022**		− 0.0002*		0.00015		0.00015	
	(− 2.03)		(− 1.91)		(1.63)		(1.59)	
Experience	− 0.9***				1.1***			
	(− 4.35)				(4.65)			
# Firms following	− 0.22***				0.35***			
	(− 20.05)				(28.42)			
Constant	− 2.5***		0.14***		4.1***		− 0.098**	
	(− 7.44)		(2.82)		(10.65)		(− 2.26)	
Visit FE	Yes		No		Yes		No	
Analyst FE	Yes		No		Yes		No	
Firm FE	No		Yes		No		Yes	
Analyst-year FE	No		Yes		No		Yes	
*N*	802		818		802		818	
*R*^2^	0.655		0.654		0.790		0.790	

Continuous variables are winsorized at the 99th percentile. T-statistics are displayed in parentheses. Robust SEs are clustered at the firm level. Statistical significance *p < 0.1, **p < 0.05, ***p < 0.01.

Compared with the previous US-based study^9^, the moderation effect of surname commonness seems to be weaker in our Chinese sample, as the effect of surname match on forecast accuracy (Columns 1 and 2) do not differ between common and uncommon matched surnames. In contrast, prior U.S. research^9^ found that surname rarity plays a more substantial role in moderating forecast accuracy. This difference indicates that surname-based biases are less influenced by surname rarity in China, highlighting the cultural specificity of these effects, where surname matching remains impactful regardless of commonness, potentially driven by prevalent cultural values like *Mianzi*.

### Self-selection issue

**Fig. 2:**
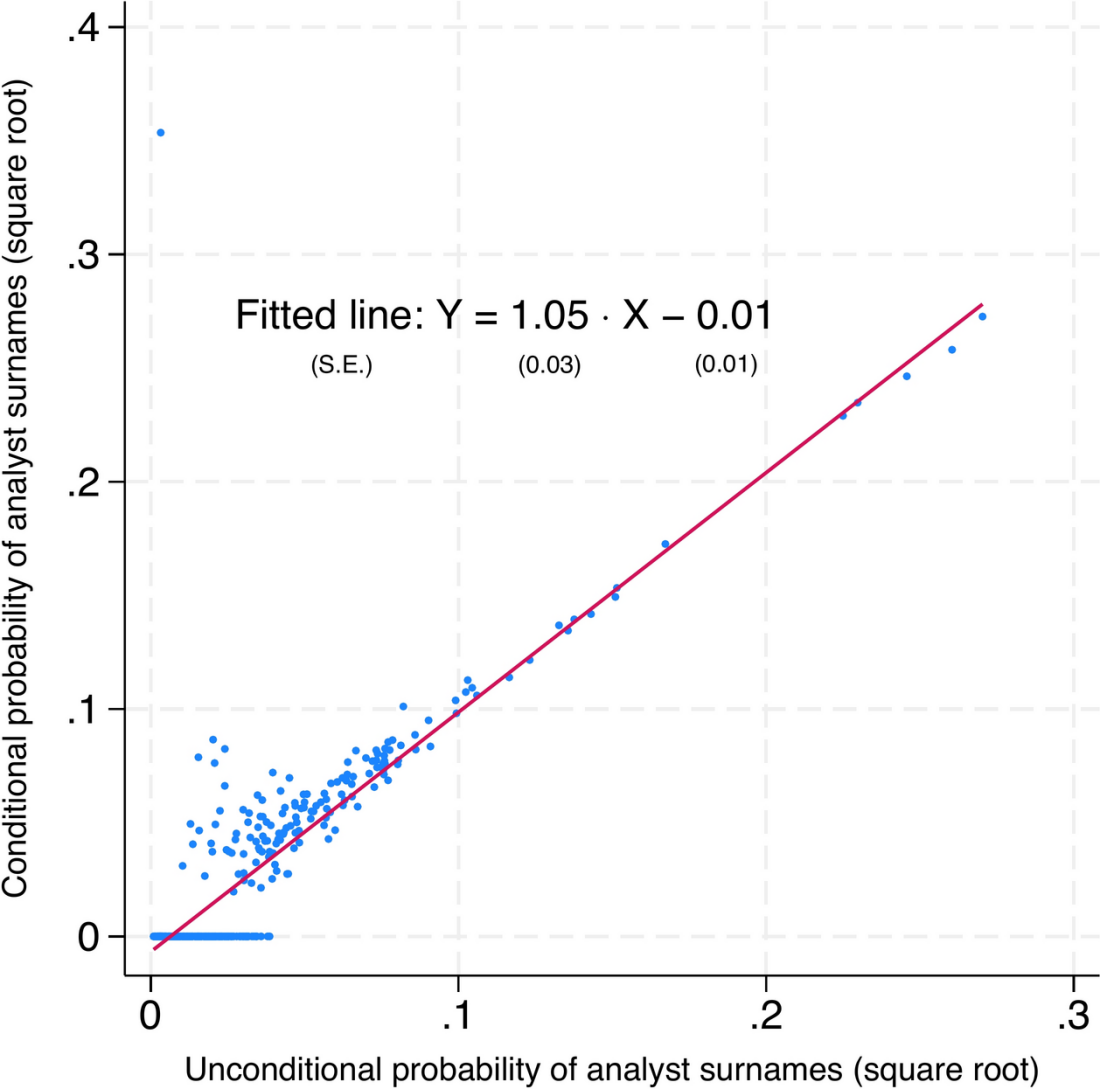
Testing the empirical conditional probability of surnames against potential self-selection.

One issue that could invalidate our results is that firms may only respond to visit requests made by analysts who are likely to issue an optimistic forecast, and analysts may self-select to cover certain firms^43^. To test this, we check whether surname-matched analysts are more likely to happen during a corporate site visit, either due to a firm’s incentive to curry favor from the visiting analysts or due to the analysts’ increased willingness to visit a firm. Our analysis (Fig. 2) finds no systematic difference in the likelihood of treated and control analysts visiting a firm, regardless of their surnames. This validates our assumption that the willingness of analysts to visit a firm could indicate a relatively uniform level of information advantage across the visiting analysts. It is a rational assumption the visited firm unlikely chooses someone based on the surname to please one specific analyst among teams of visitors^9^. We also performed a t-test on whether the visiting analysts’ attributes differ in terms of whether they share a surname with the representative or not. Those are not significant; therefore, we deem it unlikely that such reverse causality exists. Overall, we find no systematic impact of corporate representative surnames on the distribution of the analyst attributes. Hence, our findings should not be driven by surname sorting between corporate representatives and analysts, but rather, by incidental surname matching.

Our results collectively suggest that while incidental surname matching led to more accurate forecasts in the US^9^, this may skew analyst judgments towards optimism in a different cultural context. This bias is more pronounced for analysts with uncommon surnames. Although stronger evidence for cultural differences would come from a single investigation with data from both countries, our findings provide valuable nuance to existing research and underscore the need for a deeper understanding of the implications of incidental similarities.

## Discussion

Having established that surname matching leads to biased optimism for Chinese analysts, we now discuss the broader cultural implications of these findings and their relevance to market regulation. The findings of this study illustrate the enduring influence of culturally specific values like *Mianzi*. Cultural evolutionary theory^29,30^ suggests that such values are shaped by historical contexts and continue to affect social behaviors today. Our results suggest that *Mianzi*, as a culturally constructed form of social reputation, plays a role in how surname matching influences financial analysts’ forecasts in China. These insights underscore how cultural context differentiates Chinese professional decision-making from Western norms emphasizing autonomy or self-determination^31^. Future research could conduct a single investigation using data from both countries, offering more robust evidence regarding the cultural specificity of name-matching biases. In the present study, the analysts visiting the same firm on the same occasion constitute a relatively homogeneous group in terms of socioeconomic and educational background. However, future research should also investigate whether such socioeconomic factors may play a role during face-to-face interactions in a more diverse population. Additionally, further studies could extend this exploration to other Trans-Himalayan and Altaic language-speaking countries.

The results also contribute to academic research on analysts’ earnings forecasts by demonstrating a strong positive relationship between the analyst’s forecasting optimism and the surname homophily with the corporate representative hosting the site visit. Specifically, we answer the call for more research on the impact of private meetings on forecasting accuracy^44,45^. This line of literature has been scant potentially due to the lack of disclosure for private communications. As noted by researchers, China-related research on in-person interactions between the management and the analysts significantly moves the field forward^46,47^. The mandatory disclosure of site visits to listed firms in China provides a representative natural laboratory. The extant literature suggests that site visits provide an informational advantage to visiting analysts, compared to their non-visiting counterparts^11,48–50^. Forecasting accuracy, in general, is higher for visiting versus non-visiting analysts. A caveat to note here is that analysts may self-select to visit firms where they can gain more informational advantages^43,51^. By controlling for the visit fixed effect, our study compares the forecasting accuracy of analysts who share the same surname with the firm’s management and those who do not. Our study has a prolonged sample from 2011 to 2019. Combined with the extant literature, analysts obtain informational advantages during site visits, but these advantages are limited by behavioral biases.

In addition, our findings speak to the behavioral economics literature on professional judgements of financial analysts^52,53^. Specifically, we focus on favoritism bias resulting from incidental surname matching found during in-person interactions. Although social psychologists have offered much evidence of how sharing of names may alter personal behavior, applications of this theory in finance and accounting settings remain scant. Recently researchers have examined the name effect among board directors^54^ and between the auditor and the CEO^32^. We are among the first to investigate the role that incidental matching in names may play in analyst forecasts. The availability of site visit records in China enables us to track private interactions among analysts and executives. We find that with the existence of in-person interactions, behavioral bias will significantly hinder the accuracy of financial information.

The findings of this study carry substantial policy implications for regulatory bodies and financial institutions around the world. Even though personal affiliations manifest in different ways, they inadvertently affect the perceived integrity of financial analyses, whether in the US or in China. This suggests a need for policy frameworks that encourage transparency and objectivity in analyst-corporate interactions. Regulators may consider implementing guidelines for disclosure that specifically address the potential for unconscious biases, ensuring that financial forecasts are as impartial as possible. Moreover, policies that foster diverse and collaborative analytical processes could serve as a counterbalance to individual biases, as evidenced by the moderation of initial optimistic forecasts in later revisions and when reports are prepared with co-authors.

For firms and professional bodies, these insights emphasize the importance of cultivating an awareness of implicit biases in their training and professional development programs. It would be prudent for financial firms to establish protocols that encourage critical review processes, especially for forecasts and analyses that may be influenced by analysts’ personal connections and similarities. These measures, however, should take into account the cultural distinctions. For example, to prevent private information sharing in the US^9^, possible measures could include rotating analysts for company visits. But to avoid over-optimism that Chinese analysts exhibit, firms can assign teams rather than individuals to analyze a particular company. Additionally, continued investment in training programs that focus on unconscious bias could help analysts recognize and adjust for personal predilections, potentially enhancing the accuracy and reliability of their reports. By acknowledging and addressing these psychological factors, the financial industry can work towards more robust and trustworthy analytical practices that better serve the interests of all market participants.

## Supplementary Information

Supplementary Information.

## Data Availability

The data supporting the findings of this study are available from CSMAR. Access to the data requires a subscription on the CSMAR platform. Researchers with such subscriptions can obtain the raw data directly from CSMAR.
