# Supplementary material for: The effect of incidental name similarity on favoritism in the Chinese financial market
Source: Sci Rep. 2025 Apr 16;15:13077. doi: 10.1038/s41598-025-97364-x (PMC12003653; doi:10.1038/s41598-025-97364-x)
Supplement: Supplementary file 1 — Supplementary Information. [file 41598_2025_97364_MOESM1_ESM.docx]

**Supplementary Material**

for

**The Effect of Incidental Name Similarity on Favoritism in the Chinese Financial Market**

Kaixian Mao, Huidi Lu & Shirley Jiexuan Wang

**Part SM1. Summary Statistics**

Table SM1 Panel A. Variable Summary Statistics for the Primary Study (Study 1) Based on Site Visits

|  | | Mean | SD | Min | Max | Correlations | | | | | | |
| --- | --- | --- | --- | --- | --- | --- | --- | --- | --- | --- | --- | --- |
|  |  |  |  |  |  | 1 | 2 | 3 | 4 | 5 | 6 | 7 |
| 1 | RFA | -0.10 | 0.60 | -2.33 | 1 | 1 |  |  |  |  |  |  |
| 2 | ROPT | 0.10 | 0.69 | -1.84 | 2.29 | -0.57 | 1 |  |  |  |  |  |
| 3 | Surname match | 0.37 | 0.48 | 0 | 1 | -0.06 | 0.05 | 1 |  |  |  |  |
| 4 | Forecast horizon | 457.30 | 279.71 | 0 | 1019 | -0.17 | 0.10 | 0.00 | 1 |  |  |  |
| 5 | Experience | -1.78 | 7.10 | -14.38 | 23.49 | -0.04 | 0.02 | 0.02 | 0.00 | 1 |  |  |
| 6 | # Firms following | -7.16 | 18.13 | -45.14 | 60.5 | -0.03 | -0.02 | -0.05 | 0.03 | 0.64 | 1 |  |
| 7 | Uncommon surname | 0.29 | 0.45 | 0 | 1 | 0.07 | -0.04 | -0.39 | -0.03 | -0.09 | 0.05 | 1 |

N = 935

Table SM1 Panel B. Variable Summary Statistics of the Full Analyst Forecast Sample (Study 2)

|  | | Mean | SD | Min | Max | Correlations | | | | | | |
| --- | --- | --- | --- | --- | --- | --- | --- | --- | --- | --- | --- | --- |
|  |  |  |  |  |  | 1 | 2 | 3 | 4 | 5 | 6 | 7 |
| 1 | RFA | 0.07 | 0.58 | -2.04 | 1 | 1 |  |  |  |  |  |  |
| 2 | ROPT | -0.06 | 0.68 | -1.80 | 2.21 | -0.55 | 1 |  |  |  |  |  |
| 3 | Surname match | 0.30 | 0.46 | 0 | 1 | 0.00 | 0.01 | 1 |  |  |  |  |
| 4 | Forecast horizon | 413.89 | 301.98 | 6 | 1023 | -0.19 | 0.12 | 0.01 | 1 |  |  |  |
| 5 | Experience | -0.18 | 7.88 | -14.34 | 26.37 | 0.01 | -0.01 | 0.00 | -0.01 | 1 |  |  |
| 6 | # Firms following | -1.05 | 28.50 | -65.33 | 139.67 | 0.00 | 0.00 | 0.01 | 0.00 | 0.55 | 1 |  |
| 7 | Uncommon surname | 0.35 | 0.48 | 0 | 1 | 0.01 | -0.01 | -0.41 | 0.00 | -0.01 | -0.02 | 1 |

N = 399,759

**Part SM2. Robustness Checks on Incidental Similarities Among Uncommon Surnames**

Following Even-Tov et al. (2023), we define the indicator variable *Uncommon* based on selected thresholds and test how surname uncommonness moderates the relationship between surname similarity and analyst forecasting biases. For example, a 0.5% threshold classifies surnames that account for less than 0.5% of the population as uncommon. We opted for 0.5% in the main analysis instead of the 2% threshold used in Even-Tov et al. (2023) because the distribution of Chinese surnames differs significantly from that of U.S. first names. Applying a 2% threshold would classify all but the top 7 most common Chinese surnames as uncommon, leading to the misclassification of genuinely common surnames as uncommon. This misclassification would weaken the observable effect and hinder our ability to detect its true impact.

To ensure robustness, we tested thresholds of 0.7%, 0.5% (main result), 0.3%, and 0.1% using the same interaction model specified in Part SM4 in this Supplementary Material. Table SM2 shows that stricter thresholds lead to more significant effects (e.g., *p* < 0.05 in Columns 6 and 8 versus *p* < 0.1 in Columns 2 and 4). These results are consistent with our expectations.

Table SM2. Effect of Surname Match Moderated by Commonness Using Different Thresholds

|  | (1) | (2) | (3) | (4) | (5) | (6) | (7) | (8) |
| --- | --- | --- | --- | --- | --- | --- | --- | --- |
| DV: ROPT | Threshold = 0.7% | | Threshold = 0.5% | | Threshold = 0.3% | | Threshold = 0.1% | |
| Surname match | 0.45*** | 0.31*** | 0.36* | 0.31*** | 0.50*** | 0.31*** | 0.50*** | 0.31*** |
|  | (3.61) | (18.36) | (1.89) | (18.35) | (16.02) | (18.36) | (16.03) | (18.36) |
| Surname match × Uncommon | **0.26**** | **0.23*** | **0.34**** | **0.30*** | **0.25**** | **0.25**** | **0.27**** | **0.28**** |
|  | **(2.38)** | **(1.88)** | **(2.22)** | **(1.94)** | **(2.15)** | **(2.13)** | **(2.14)** | **(2.12)** |
| Forecast horizon | .00015 | .00015 | .00015 | .00015 | .00015 | .00015 | .00015 | .00015 |
|  | (1.63) | (1.59) | (1.63) | (1.60) | (1.62) | (1.58) | (1.62) | (1.58) |
| Experience | 1.24*** |  | 1.12*** |  | 1.30*** |  | 1.30*** |  |
|  | (7.80) |  | (4.65) |  | (32.93) |  | (32.94) |  |
| # Firms following | 0.35*** |  | 0.35*** |  | 0.36*** |  | 0.36*** |  |
|  | (42.69) |  | (28.42) |  | (111.03) |  | (111.02) |  |
| Constant | 4.31*** | -0.07 | 4.12*** | -0.06 | 4.42*** | -0.09* | 4.42*** | -0.09* |
|  | (17.21) | (-1.42) | (10.65) | (-1.12) | (55.05) | (-1.95) | (55.41) | (-1.94) |
| Visit FE | Yes | No | Yes | No | Yes | No | Yes | No |
| Analyst FE | Yes | No | Yes | No | Yes | No | Yes | No |
| Firm FE | No | Yes | No | Yes | No | Yes | No | Yes |
| Analyst-Year FE | No | Yes | No | Yes | No | Yes | No | Yes |
| *N* | 802 | 818 | 802 | 818 | 802 | 818 | 802 | 818 |
| *R*^2^ | 0.790 | 0.790 | 0.790 | 0.790 | 0.790 | 0.790 | 0.790 | 0.790 |

Notes: Continuous variables are winsorized at the 99th percentile. T-statistics are displayed in parentheses. Robust SEs are clustered at the firm level. Statistical significance * *p* < 0.1, ** *p* < 0.05, *** *p* < 0.01.

**Part SM3. Experimental Evidence on the Different Social Implications of Surname Match**

We conducted an online survey to explore the different consequences associated with surname similarity among Chinese and American participants. This result is both important in motivating our paper and in providing additional proof of cultural specificity in surname matching effect. As described in the main text, we administered an online survey using a 2 (nationality: Chinese vs. American) × 2 (surname match vs. no surname information) between-subjects design. We surveyed 220 Chinese nationals on Credamo and 220 US nationals on Prolific (*N* = 432 in total after excluding participants who failed the attention check). In this survey, participants were randomly assigned to either the surname match condition, where they are told that they just met a person of the same surname as theirs, or to the no information condition, where surname of the other person was not mentioned. We measure perceived connection towards another individual using a set of four questions: 1) How likely do you think you two are relatives? [Related] 2) How similar do you think your values might be? [Values] 3) How willing would you be to exchange contact information with this person? [Contacts] 4) How likely do you think it is that you two could become good friends with each other? [Befriend]. Each question is measured on a 1-5 Likert scale, with 1 being the least likely/willing and 5 being the most. We regress each dependent variable on an indicator of being assigned to the surname match condition, an indicator of the Chinese sample, and their interactions. We include gender and higher education dummies as control variables. Table SM3 presents the regression results.

Table SM3. The Social Implications of Surname Match in Chinese vs. American Individuals

|  | (1) | (2) | (3) | (4) |
| --- | --- | --- | --- | --- |
|  | DV: Related | DV: Values | DV: Contacts | DV: Befriend |
| Surname match | 0.94*** | -0.24* | -0.13 | -0.15 |
|  | (5.81) | (-2.45) | (-0.86) | (-1.38) |
| Chinese | 0.12 | -0.19* | 0.01 | -0.14 |
|  | (0.92) | (-2.05) | (0.09) | (-1.24) |
| **Surname match × Chinese** | **-0.33** | **0.27*** | **0.62**** | **0.57***** |
|  | **(-1.66)** | **(2.10)** | **(3.16)** | **(3.57)** |
| Female | -0.04 | -0.12 | -0.17 | -0.15 |
|  | (-0.34) | (-1.67) | (-1.66) | (-1.72) |
| Higher education | -0.17 | 0.10 | -0.18 | -0.10 |
|  | (-1.03) | (0.86) | (-1.18) | (-0.76) |
| Constant | 1.83*** | 3.28*** | 3.42*** | 3.39*** |
|  | (10.64) | (24.63) | (20.64) | (26.17) |
| *N* | 432 | 432 | 432 | 432 |
| *R*^2^ | 0.139 | 0.027 | 0.054 | 0.046 |

*Notes*: T-statistics are in parentheses. Statistical significance: * *p* < 0.05, ** *p* < 0.01, *** *p* < 0.001

**Part SM4. Alternative Test on the Moderating Effect of Surname Commonness**

To study the moderating effect of surname commonness, we followed the literature and constructed two variables capturing surname match for common and uncommon names separately. In this section, we use an alternative test to illustrate the robustness of the effect. Specifically, we include an interaction term between surname match and commonness of the analyst’s surname. Table SM4 shows the regression results. We see the same pattern as our main analysis in Table 3, that uncommon surname match further increases the name match effect on analyst forecast optimism (Column 3 and 4). To ensure robustness, we tested thresholds of 0.7%, 0.5% (main result), 0.3%, and 0.1% to define the uncommon surname indicator. The robustness check results are shown in Part SM2.

Table SM4. Effect of Surname Match Moderated by Commonness

|  | (1) | (2) | (3) | (4) |
| --- | --- | --- | --- | --- |
|  | RFA | RFA | ROPT | ROPT |
| Surname match | -1.46*** | -0.36*** | 0.36* | 0.31*** |
|  | (-8.87) | (-24.36) | (1.89) | (18.35) |
| Surname match × Uncommon | **0.20** | **-0.08** | **0.34**** | **0.30*** |
|  | **(1.45)** | **(-0.35)** | **(2.22)** | **(1.94)** |
| Forecast horizon | -.00022** | -.0002* | .00015 | .00015 |
|  | (-2.03) | (-1.91) | (1.63) | (1.60) |
| Experience | -0.90*** |  | 1.12*** |  |
|  | (-4.35) |  | (4.65) |  |
| # Firms following | -0.22*** |  | 0.35*** |  |
|  | (-20.05) |  | (28.42) |  |
| Constant | -2.46*** | 0.09* | 4.12*** | -0.06 |
|  | (-7.44) | (1.68) | (10.65) | (-1.12) |
| Visit FE | Yes | No | Yes | No |
| Analyst FE | Yes | No | Yes | No |
| Firm FE | No | Yes | No | Yes |
| Analyst-Year FE | No | Yes | No | Yes |
| *N* | 802 | 818 | 802 | 818 |
| *R*^2^ | 0.655 | 0.655 | 0.790 | 0.790 |

Notes: Continuous variables are winsorized at the 99th percentile. T-statistics are displayed in parentheses. Robust SEs are clustered at the firm level. Statistical significance * *p* < 0.1, ** *p* < 0.05, *** *p* < 0.01.
